# Prevalence of traumatic injuries in deciduous dentition and associated risk factors in a Spanish children population

**DOI:** 10.4317/jced.58051

**Published:** 2021-07-01

**Authors:** Beatriz Prieto-Regueiro, Gladys Gómez-Santos, Montserrat Diéguez-Pérez

**Affiliations:** 1 Primary Care Odontostomatologist. Health Services Management of the Health Area of ​​Lanzarote. Canary Health Service; 2 Stomatologist. Technician of the General Directorate of Public Health. Health Promotion Service. Canary Health Service; 3 Adjunct Professor. Department of Preclinical Dentistry. Faculty of Biomedical Sciences and Health Sciences. European University of Madrid. Associate Professor of Pediatric Dentistry. Department of Dental Clinical Specialties. Faculty of Dentistry. Complutense University of Madrid

## Abstract

**Background:**

The frequency of traumatic dental injuries (TDI) in primary dentition and those agents that favor them present a great variability. Therefore, the objective of this study was to determine the prevalence of TDI in a population of Spanish preschoolers in temporary dentition and identify the factors associated with these injuries.

**Material and Methods:**

An epidemiological observational prevalence study was carried out. A total of 343 children aged between 3 and 5 years were selected. A questionnaire was completed in relation to socioeconomic factors, cultural level, dietary habits and oral parafunction. Through an intraoral examination, information was collected on the occlusal characteristics and the presence of TDI. Data analysis was performed using the SPSS Statistics 25.0 program for Windows, using the descriptive and frequencies procedure, contingency tables, Chi-square test, and logistic regression analysis.

**Results:**

The prevalence of TDI in the total sample was 12.2%. The most frequent lesion was crown discoloration (0.4%) followed by crown fracture (0.1%) and avulsion (0.1%). The most affected tooth was the deciduous upper central incisor. In the multivariate logistic regression analysis the presence of dental trauma did correlate significantly with the age (5 years in reference to 3 years: OR = 4.209; 95% C.I. = 1.591-11.134; *p* = 0.004) and overjet (OR = 2.609; 95% C.I. = 1.306-5.214; *p* = 0.007).

**Conclusions:**

The prevalence of these lesions in a Spanish infant population with temporary dentition is low. Only age and overjet are risk factors.

** Key words:**Dental trauma, deciduous dentition, risk factors.

## Introduction

Oral health during childhood can often be altered by both caries and traumatic injuries. The clear decrease in the prevalence of the former, evidenced by epidemiological studies in developed countries, suggests that dental trauma could become the main reason for consultation in pediatric dental practice in the future. The frequency of these traumatic dental injuries (TDI) in children represents a great variability between countries and study populations. This is influenced in part by the presence of biological, behavioral, cultural, social, human, environmental and economic risk factors. The number of known causes has increased to unsettling levels, probably due to the greater interest in them. During the first years of life, when the child begins to crawl, walk, run and later when he performs sports and recreational activities, the appearance of these accidents is favored; the home, the school and the recreational parks being the places where they usually occur. The infant’s physical, social and emotional well-being is directly linked to his personal relationships and his self-esteem (1-6).

This type of trauma constitutes a problem for public health and entails significant expenses, as well as long follow-up periods. All of this implies the need to plan prevention strategies aimed above all at the identification, understanding and control of risk factors. The evaluation of the trends of a disease helps public authorities to determine the needs of the child population effectively and to promote preventive strategies, also to plan and structure health services. By requiring immediate attention, campaign planning should be addressed to parents, educators, children and primary care health personnel, thus favoring not only their early treatment but also their prevention. We can not ignore that, in childhood, a correct diagnosis can intercept the appearance of complications after the treatment of these TDIs (2-4,7-19).

In recent years, studies on prevalence and risk factors have been much more numerous in children and / or adolescents with mixed or permanent dentition, and if we refer to the Spanish population, they have been scarce. The factors associated with these lesions in the primary dentition have been investigated, but the real knowledge with respect to most of these variables remains uncertain (8-26).

Therefore, the objective that we set out in our research was to determine the prevalence of TDI in a population of Spanish preschoolers in temporary dentition and identify the factors associated with these injuries.

## Material and Methods

An observational, descriptive, cross-sectional or prevalence epidemiological study was carried out that complies with the ethical precepts formulated in the Declaration of Helsinki, the Patient Autonomy Law and the Organic Law on Data Protection. The Ethics Committees of the European University of Madrid and the Maternal and Child Hospital of Las Palmas de Gran Canaria approved the study protocol, reference number CIPI/028/15 and CEIC-CHUIMI-2015/807 respectively.

 The fieldwork has been carried in Arrecife, municipality of Lanzarote Island, randomly selected. The sample size and precision for the estimation of a population size of 2108, with an expected proportion of 70%, 95% confidence level, design effect 1, an accuracy of 5%, was 283 patients, however has expanded the sample to 343.

A only calibrated explorator randomly selected patients who attended a primary care pediatrician visit.

The control of intraobserver variability was carried out with duplicate examinations, reexploration to 10% of the sample in a period of less than 1 week between the first and the second revision, whose kappa index has been between 0.9 and 1, which, according to the scale of Landis and Koch, situates the intra-examiner agreement in the maximum range of degree of agreement (0.81-1.00).

The inclusion criteria involved patients of both sexes, from 3 to 5 years of age, owners of the Canary Health Card whose parents signed the informed consent. The exclusion criteria included the refusal to participate at any time during the study and the lack of collaboration of the children in the dental exploration.

For the collection of data, a questionnaire and dental examination form were used. The time dedicated to the interview was 10 to 15 minutes. Seven patients were cited per day and, following the WHO (World Health Organization) 2013 criteria, for the oral examination an intraoral plane mirror of number 5 was used and it was carried out with the preschool child sitting in the dental chair with the neck in extension and the researcher standing and behind him. Depending on the degree of collaboration, the estimated time for each patient was 10 to 20 minutes.

The variables used in the questionnaire, have been obtained through a directed and personalized interview questions to the preschoolers´parents. The abbreviated classification of the Working Group of the Spanish Society of Epidemiology and the Spanish Society of Family and Community Medicine (27) was used for the evaluation of the social class, and the level of studies. To measure social class, we relied on the occupation that the head of the family played at the time of study or has played previously (in the case of retired or unemployed), understanding this Figure as the person who contributed most to the family budget regularly. The level of maternal studies served as a family cultural indicator, since mothers are present in almost all families and this Figure is more influential in the health of children. When the father was the only adult in the home, his level of education was taken into account. A low level was considered if the mother was illiterate, without studies or with only primary studies; medium level if she had secondary school diploma; and high level studies with university studies. Due to the intensification of the phenomenon of immigration in the study area, it was decided to register the child’s origin. It was considered of Spanish nationality when both parents were of said origin, and of foreign nationality if one of them was from another country. If this information was unknown, at least one of the parents was considered as an unknown source. The last variable included in the questionnaire was parafunctional habits (pacifier use, digital suction, onicofagia, bruxism, lip incompetence). It was considered a prolonged use of the pacifier, or digital suction when the preschool has used it for a period equal or longer than 2 years and considered a current use if the child also continued with this habit at the time of the study. The occlusal characteristics according to the three planes of space were reflected in the dental examination form. In the anteroposterior plane the Terminal planes and the overjet were studied; in the transverse plane, the crossbite; and in the vertical plane, the overbite and openbite. For the study of the TDI (crown discoloration, crown fracture and avulsion) and its distribution by dental unit, the WHO 2013 criteria was followed. These variables were also collected in the dental examination form.

The statistical analysis of the data was done through the SPSS 25.0 program for Windows. A descriptive statistics of quantitative variables (descriptive procedure) and for the qualitative variables (frequencies procedure), as well as contingency Tables for the relationship between qualitative variables. The Chi-square test was applied to contrast the independence or influence between two qualitative variables. A logistic regression analysis was conducted to measure the strength of the association between the independent variables age and overjet, and the dependent variable dental trauma. A value of *p* <0.05 was considered statistically significant.

## Results

The total sample of the study was of 343 patients, after applying the selection criteria; there were 18 losses, of which 5 were motivated by absence to a concerted appointment for interview and 13 due to refusal or lack of collaboration of the preschool in the exploration. The mean age (± standard deviation) of the population was of 3.27 ± 0.28, 4.39 ± 0.30 and 5.39 ± 0.33 respectively. The sample obtained was weighed by sex, 175 (51%) patients were boys and 168 (49%) girls. In terms of age, 99 preschoolers were 3 years old, 122 were 4 years old and 122 were 5 years old.

The prevalence of TDI in the total sample was 12.2%.

A higher frequency was observed in preschoolers of 5 years compared to the lowest registered in those of 3 years of age. This result was significant to *p* value <0.05 (Table 1).

**Table 1:**
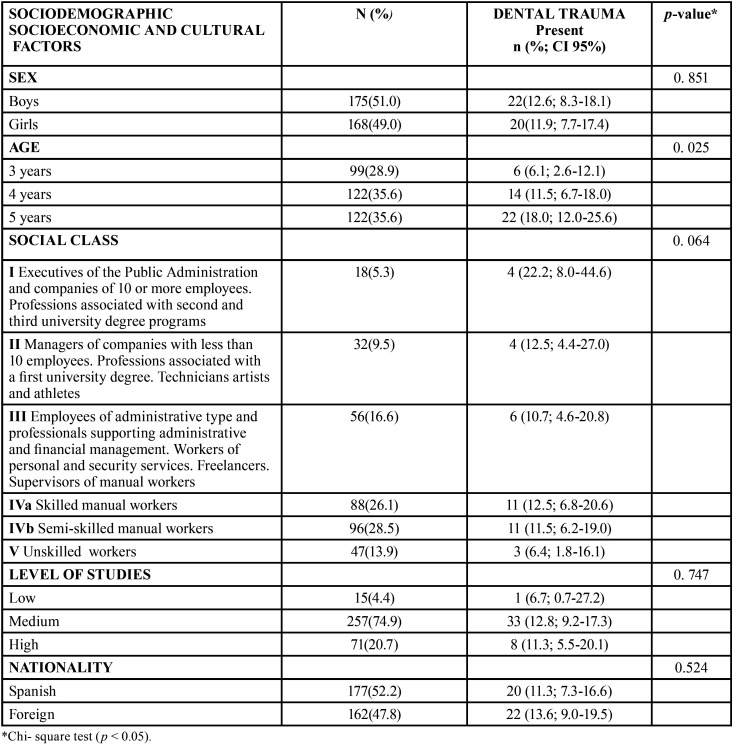
Prevalence of traumatic dental injury according to socioeconomic, cultural and nationality variables.

When studying the relationship between dental trauma and socioeconomic, we observed that the 56.4% of preschools with dental trauma corresponded to semi-skilled and skilled manual workers. In six of the patients of the total sample, their parents were unemployed or retired and only in four patients of the total sample the origin did not appear (Table 1).

The tooth that usually presented trauma was the deciduous superior central incisor. Pearson’s Chi-square indicated the existence of significant differences at 95% for p≤0.001 among the dental groups for all ages (Table 2).

**Table 2:**
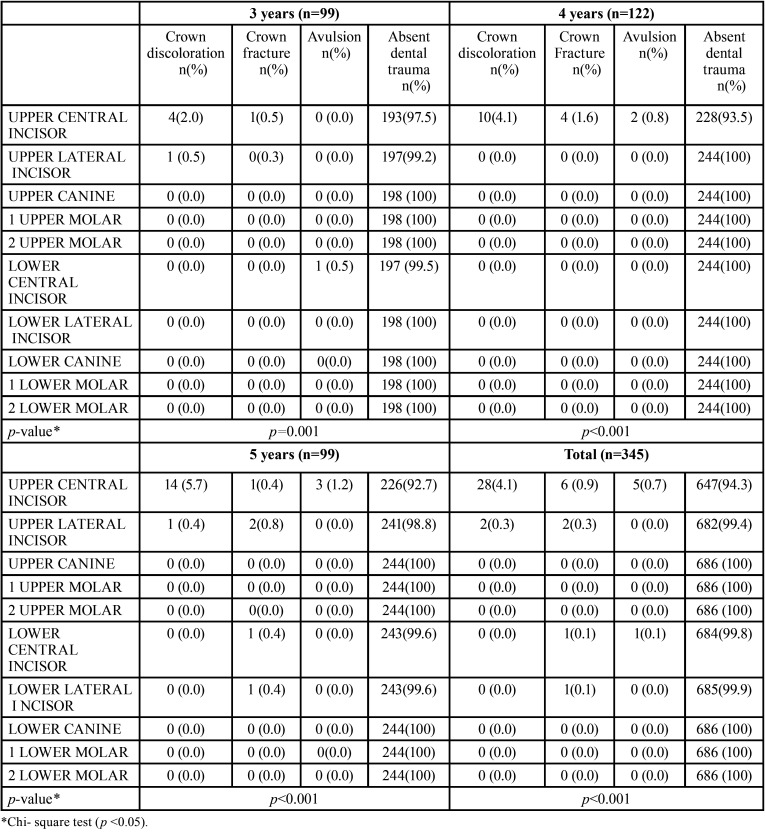
Distribution of traumatic injuries in deciduous dentition, by dental unit, age range in and the total sample.

By relating the presence or absence of dental trauma with oral parafunctional habits, the results obtained were similar. No child with a history of dental trauma was using the pacifier at the time of the study and bruxism was the most frequent parafunction. The study of occlusal factors revealed that the highest percentage of patients with a history of dental trauma is related to overjet. (Table 3).

**Table 3:**
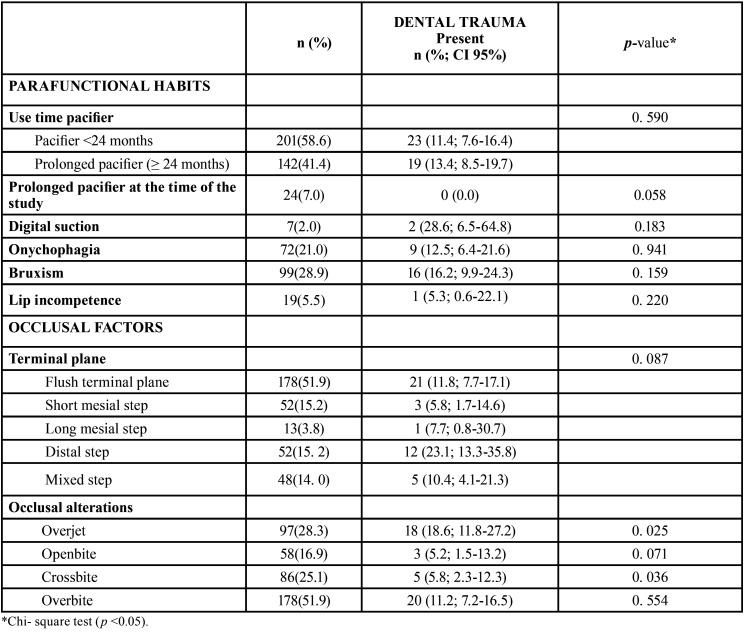
Prevalence of traumatic dental injury according to parafuntional habits and occlusal variables.

The probability of having dental trauma increases with age and with overjet. Multivariate logistic regression analyse of the influence of the independent variables age and overjet, on the dependent variable dental trauma,is presented in the Table 4.

**Table 4:**
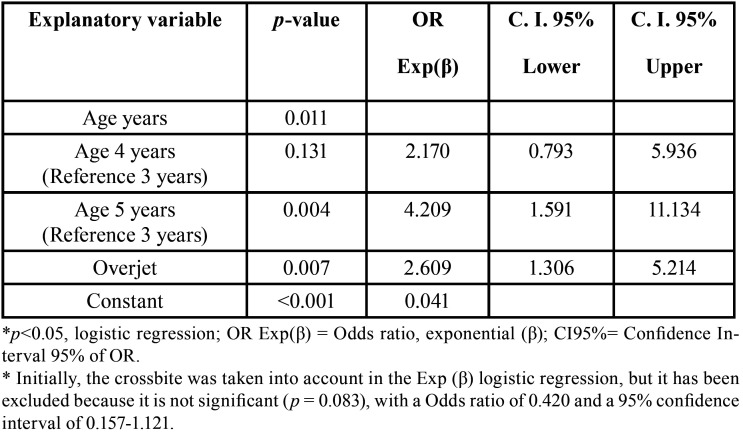
OR Main outcomes associated to patients with dental trauma.

## Discussion

The percentage of TDI in children with primary teeth is very variable from one study to another (1,2,5,12-15,17,18,22-24,26). The lowest prevalence (9.4%) was observed in Brazil in preschool children from 5 to 59 months of age (17,26). This value, somewhat lower than ours (12.2%), may be determined by the fact that the age intervals are wider than this of our patients (36-60 months).

The highest observed prevalence was 53.4% in a Brazilian cohort of 5 years of age (2). We agree with the majority of the authors that the frequency of these injuries is related to the increase in age (6,8,12,15,17,23,24,26), and differences obtained in relation to sex are significant (5,7,10,12,13,16-18,23,26). Some of the studies show that boys are 1.67 times more likely to suffer dental trauma than girls (6). And few authors have found a significant relationship between the prevalence of these traumatic injuries and gender (1,3,9,15).

If we consider the tooth that most frequently presents the TDI we agree with the majority of the studies (6,8-12,15-18,22-26) since the upper central incisors are the primary teeth more traumatized. When studying the type of lesion that presents at the dental level, for some authors (1,26) the dental discoloration is the most prevalent, a fact that we also observed in our study. For other researchers, enamel fractures (49.7%, 37.2%, 35.0%) (6,12,15,16) or crown (11.0% and 60.0%) (24,25) are the most common lesions, followed by crown discoloration (33.0%) (15). However, in other studies and unlike the results obtained in our research, this alteration is the least frequent (4.0%) (24).

We agree with the results of most of the investigations in the fact that socioeconomic factors are not associated statistically and significantly with the occurrence of TDI (1,5,7,10,12,15,17,26). However, some authors observe the significance between these injuries and the variable schooling of the parent, and children of mothers or fathers with low levels of schooling have a higher prevalence of presenting TDI (1,15). Occasionally a significant association is observed between the economic factor and dental trauma, since in these cases, the lower salary of the parents favors the higher prevalence of these lesions (24). If we take into account the origin of the parent, unlike our results, some authors observe significance when one or both parents are foreigners (7).

When studying parafunctional habits, we found zero significance between the association of dental trauma and the presence of non-nutritive suction habits, as well as other studies (18,23). If we take into account the occlusal characteristics of the infant patient in the primary dentition in some investigations (5,26), they determine how the presence of anterior open bite is related to the TDI, presenting these patients twice as likely to present these lesions. For other authors this association is statistically significant (8,15,17,21,23) and not only in the presence of an open bite but also in cases with a lack of labial sealing. However, as in our study, there is a lack of significance between labial incompetence and TDI (18) in other studies. We have determined, as other researchers, that age (3,8) and increased overjet (3,5,10,17,21,23), pose a risk to traumatic dental injury, Table 4. The statistical analyzes collected confirm, in contrast to the results obtained by us, that factors such as overbite are significant when associate with TDI (8,15,21,23).

In Spain we have only found one study similar to ours, in which the prevalence was higher (21.7%). The age range of the patients is wider (1 to 6 years). Children 1-3 years old presented these lesions more frequently, however, according to our results, the prevalence of these lesions increases with age. In relation to sex, males present more dental trauma in all age groups, except in the 3-year-old group, where, unlike our study, girls predominate. We only agree with this investigation in the fact that the primary teeth affected most frequently are the upper central incisors (86.9%) (22).

## Conclusions

The prevalence of TDI in Spanish children with primary dentition is low (12.2%) taking into account the wider range of age of other studies. The analysis of variables such as age, sex, socioeconomic and cultural factors, nationality and parafunctional habits, as well as the occlusal characteristics, determine that only the age and the overjet are considered risk factors for these injuries.
